# Current limitations in technology-based cognitive assessment for severe mental illnesses: a focus on feasibility, reliability, and ecological validity

**DOI:** 10.3389/fnbeh.2025.1543005

**Published:** 2025-04-07

**Authors:** Edoardo Caporusso, Antonio Melillo, Andrea Perrottelli, Luigi Giuliani, Francesco Flavio Marzocchi, Pasquale Pezzella, Giulia Maria Giordano

**Affiliations:** Department of Mental and Physical Health and Preventive Medicine, University of Campania “Luigi Vanvitelli”, Naples, Italy

**Keywords:** cognitive assessment, ecological momentary assessments, virtual reality, digital phenotyping, severe mental illness, schizophrenia, wearable devices, digital medicine

## Abstract

Cognitive impairments are frequently observed in subjects with severe mental illnesses (SMI), leading to a remarkable impact in their real-world functioning. Well-validated and gold standard instruments are available for the assessment of cognitive deficits, but different limitations should be considered, such as the need for specific training, lengthy administration times, practice effects, or reliance on subjective reports. Recent advances in digital technologies, such as ecological momentary assessments (EMA), virtual reality (VR), and passive digital phenotyping (DP), offer promising complementary approaches for capturing real-world cognitive functioning. In the current mini-review, we examine current research gaps that limit the application of these technologies, with a specific focus on feasibility, reliability and ecological validity. EMA may capture real-world functioning by increasing the number of evaluations throughout the day, but its use might be hindered by high participant burden and missing data. Furthermore, to achieve an accurate interpretation of EMA, studies should account for sampling and moment selection biases and the presence of several confounding factors. DP faces significant ethical and logistical challenges, including privacy and informed consent concerns, as well as challenges in data interpretation. VR could serve as a platform for both more ecologically valid cognitive assessments and rehabilitation interventions, but current barriers include technological and psychometric limitations, underdeveloped theoretical frameworks, and ethical considerations. Addressing these issues is crucial for ensuring that these novel technologies can effectively serve as valuable complements to traditional neuropsychological cognitive batteries.

## 1 Introduction

Cognitive deficits are a core feature of severe mental illnesses (SMI) and an important determinant of disability and impairments in real-world functioning (Sumiyoshi et al., 2019; Perrottelli et al., 2022; Vita et al., 2022a; Dragioti et al., 2023; Melillo et al., 2023; Mesholam-Gately et al., 2023; Firth et al., 2024; Giordano et al., 2024; Perrottelli et al., 2024), for which current therapies fail to provide satisfactory outcomes (Vita et al., 2018; Correll et al., 2023; Sampogna et al., 2023; Starzer et al., 2023). Among SMIs, cognitive deficits are particularly significant in schizophrenia, as they are often present even before the onset of the disorder, tend to persist during periods of clinical stability, and have a significant effect on everyday life functioning (Staal et al., 2000; Kravariti et al., 2007; Mesholam-Gately et al., 2009; Galderisi et al., 2020; Vita et al., 2022b; Handest et al., 2023). Given their clinical relevance, a reliable and feasible assessment of cognitive impairments is crucial for both clinical practice and research aimed at developing effective treatments (Hays et al., 2019; Vita et al., 2022b; Bucci et al., 2023; Melillo et al., 2024a).

Currently, assessment of cognitive functioning is mainly conducted either through performance-based cognitive tests or interviews, but both approaches have certain limitations. Pen-and-paper performance-based tests provide an objective method for evaluating cognitive deficits and gold-standard neuropsychological batteries are currently available and used in clinical practice to characterize patients’ cognitive profiles (Green et al., 2004; Yatham et al., 2010; Nuechterlein et al., 2023). However, despite their advantages, they still present different limitations. Firstly, they are susceptible to practice effects, becoming less effective in detecting changes in performance over time (Sahoo and Grover, 2022; Pezzella et al., 2024). Secondly, standardized cognitive batteries require specific training and have long administration times, thus limiting their effective implementation in routine clinical practice. Furthermore, traditional performance-based tests have been criticized for their lack of ecological validity, i.e., their ability to accurately capture and evaluate real-world cognitive functioning. Indeed, as these tests are typically conducted in controlled, clinical settings, they may not effectively capture how cognitive functioning translates to everyday life situations which involve distractions, multitasking demands, and emotional pressures (Sahoo and Grover, 2022). In addition, cognitive tests included in neuropsychological batteries are designed to isolate and assess cognitive domains individually. As a result, they do not resemble real-world cognitive challenges and tasks, which typically involve the complex interplay of multiple cognitive domains and are influenced by various factors, including, but not limited to, environmental distractors.

To address the challenges of extensive training requirements and lengthy administration times associated with traditional pen-and-paper cognitive batteries, computerized and tablet-based tests have been developed and adopted in recent decades. Computerized versions of pen-and-paper tests (Atkins et al., 2017), such as the tablet version of the Brief Assessment of Cognition in schizophrenia (BACS) and newly developed computerized batteries (Sahakian and Owen, 1992; Keefe et al., 2004; Levaux et al., 2007; Robbins et al., 2010) such as the Cambridge Neuropsychological Test Automated Battery (CANTAB) are relatively frequently used in clinical practice, providing scores comparable to pen-and-paper tests while requiring less supervision and clinical expertise (Hays et al., 2019). However, particularly when they are simple adaptations of pen-and-paper batteries, the introduction of technological versions of neuropsychological tests does not address the lack of ecological validity (Levaux et al., 2007; Feenstra et al., 2017; Harris et al., 2024). Furthermore, the absence of direct supervision and monitoring undermines the reliability of results, for instance introducing the risk of errors due to self-administration of tests (Levaux et al., 2007; Feenstra et al., 2017).

The evidence of the limited ecological validity and correlation with real-life functioning of both traditional and computerized tools prompted research efforts to develop new assessment methods. In relation to schizophrenia, within the Measurement and Treatment Research to Improve Cognition in Schizophrenia (MATRICS) initiative, the Food and Drug Administration indicated the need to integrate traditional cognitive batteries with co-primary outcomes focusing on the patients’ and caregivers’ perspectives on the real-life burden of cognitive impairment through interview-based evaluations like the cognitive assessment Interview (CAI) (Ventura et al., 2010; Cuesta et al., 2021; Bucci et al., 2023; Pezzella et al., 2024). Interview-based evaluations provide an index of how cognitive impairments impact everyday functioning in people with schizophrenia by collecting both patients’ and caregivers’ perspectives. Therefore, these assessments might detect subtle cognitive deficits that are subjectively experienced, but not captured by objective measures (Giordano et al., 2022a; Pezzella et al., 2024). Additionally, their administration requires less time and training, and their scores are not subject to practice effects, so they may more reliably detect improvements over short periods. However, as they rely on the self-reports of patients and caregivers, they can be influenced by psychopathology and biased by the individuals’ insight and capacity to assess potential cognitive difficulties (Petersen et al., 2019; Zimmerman, 2024). For instance, individuals with depressive symptoms tend to complain more about cognitive difficulties, while older subjects who experienced slow cognitive decline may be less aware of their difficulties and may have developed a series of compensation strategies in real-life (Petersen et al., 2019). Similarly, in schizophrenia, these tools can be affected by other psychopathological dimensions such as negative symptoms, which may bias both self-reported assessments and evaluations provided by caregivers (Handest et al., 2023; Rucci et al., 2023). Lastly, since interview-based assessments such as the CAI rely also on information provided by caregivers regarding the patient’s cognitive functioning, the validity of these scales is affected by variability in caregivers’ familiarity with the patient, inconsistent agreement between their evaluations and the patient’s self-assessment, and the frequent unavailability of informants (Bucci et al., 2023).

The highlighted limitations of both traditional and interview-based assessment have contributed to advancing research efforts for the development of complementary approaches. During the past two decades, recent advances in digital technologies, including ecological momentary assessments (EMA), virtual reality (VR), and passive digital phenotyping (DP), have emerged as promising tools to address the limitations of current assessment instruments. Technology-based cognitive assessment tools share the advantage that they do not require extensive training and time-consuming procedures for administration, making them more practical for both clinical and research settings. Additionally, their higher flexibility and adaptability to individual needs, as well as increasing accessibility, hold promise of delivering person-tailored assessments. Importantly, these tools have all been developed to enhance the ecological validity of cognitive assessments, either by delivering remote evaluations of real-life cognitive functioning, as in the case of EMA and DP, or by replicating real-life tasks and scenarios in virtual environments, as in the case of VR. These advantages position technology-based cognitive assessment tools as potential co-primary measures to traditional cognitive evaluations.

In the present paper, we provide a concise overview of the current state of the art of these approaches with the particular goal of highlighting current gaps currently limiting their implementation in clinical practice and research paradigms.

## 2 Methods

The present study is a mini-review aimed at synthesizing key findings on technology-based cognitive assessments in severe mental illness, identifying methodological considerations, feasibility aspects, and ethical challenges. For a more detailed description of the methodology, please refer to the Methods section of the Supplementary Material.

## 3 Results

### 3.1 Ecological momentary assessment

Ecological momentary assessment (EMA) is a method that provides real-time assessment of individuals through digital devices. EMA typically involves multiple assessments throughout the day, which take place in real-life settings and are initiated by the participant in response to alerts or push notifications at fixed or random intervals, depending on the designed paradigm (Parrish et al., 2021; Shvetz et al., 2021; Torous et al., 2021; Stone et al., 2023). By capturing data in real-world settings, EMA aims to enhance ecological validity and temporal resolution compared to traditional methods and to provide a more accurate representation of daily functioning (Smyth and Ebner-Priemer, 2025). Remote cognitive assessments using EMA via smartphones or smart watches are increasingly employed to evaluate cognition. Depending on their content, EMA paradigms of cognitive assessments can be categorized as either performance-based or interview-based. Performance-based assessments vary widely and include traditional cognitive tasks, adapted for EMA devices (Parrish et al., 2021; Shvetz et al., 2021), as well as tests specifically designed for EMA applications. Interview-based assessments, similar to traditional interviews evaluating functional capacity and cognitive performance, rely on subjective self-reports. The purported advantages of EMA, compared to traditional methods, lie in its ability to gain insights into the influence of naturalistic settings on cognitive functioning, thus delivering more ecologically valid evaluations of an individual’s cognitive functioning. In addition, EMA can achieve higher temporal resolution through multiple assessments per day, allowing it to capture intra-individual variations in cognitive performance through time (both circadian patterns and medium/long-term trajectories) (Moore et al., 2017). This approach has the potential to provide novel insights into the highly dynamic nature of cognitive functioning and the influence of various factors, including individual characteristics (e.g., mood, motivation), medication effects and environmental influences (e.g., noise or social distractors) (Moore et al., 2017; Weizenbaum et al., 2020).

However, these premises pose significant challenges. Firstly, the complex nature of EMA tools and the continuously evolving methodologies complicate the estimation and interpretation of their psychometric properties (Stone et al., 2023). Additionally, reliability estimates are not commonly reported in applied EMA research, and are limited to feasibility studies with low sample sizes (Liu et al., 2019; Moore et al., 2022; Singh et al., 2023; Stone et al., 2023; Harmon et al., 2024; Henneghan et al., 2025). Recent validation studies conducted in clinical and non-clinical populations have yielded varying results in relation to both within-person and between-person variability (Moore et al., 2022; Singh et al., 2023; Harmon et al., 2024; Henneghan et al., 2025). While lower within-person reliability—and thus higher intra individual variability— may be desirable in EMA assessment, given that its goal is to track the dynamic fluctuations in cognitive functioning, these observed changes must still reflect true and clinically meaningful variations (Stone et al., 2023). However, capturing real-world cognitive performance requires accounting for the numerous contextual variables that influence participants’ daily lives. This is necessary not only to interpret accurately the within-person variability of cognitive performances and to draw inferences on its potential causes but also to allow comparison between individuals, which is necessary for the development of normative data (Holmlund et al., 2019). Two possible examples are the environmental noise and external distractors. For instance, in the study by Hawks et al. (2023), momentary cognitive performance was worse when assessments were completed in environments perceived as noisy or busy; when participants reported difficulty concentrating before the assessment; and when participants experienced interruptions during the assessment. This is particularly problematic as a growing body of literature indicates that environmental features are often correlated with sociodemographic characteristics (e.g., noise is higher among communities with mid-to-low incomes per capita), further complicating the interpretation of results (Dreger et al., 2019; Huang et al., 2021; Hayward and Helbich, 2024). Many studies have attempted to account for environmental noise and other distractors by asking participants about their surroundings, but self-reports may be influenced by deficits in cognitive functioning. For instance, individuals with higher levels of cognitive impairments may describe their surroundings as noisier and more disruptive. Thus, subjective reports would probably need to be complemented by more objective evaluations, such as recordings of acoustic pollution or eye movement tracking, in order to detect distractions (Zamora et al., 2017; Yaroslavsky et al., 2019; Sweere et al., 2022; Hawks et al., 2023). These considerations suggest that EMA may require multifaceted data to produce evaluations that are truly ecologically valid (Weizenbaum et al., 2020).

Other current limitations of EMA depend on the involvement of multiple assessments per day to achieve higher temporal resolution and ecological validity. A first consequential challenge is related to practice effects when EMA employ performance-based tests (Hays et al., 2019), as repeated tasks may lead to improvements in performance over time. Recently, researchers have aimed to limit this effect by increasing variations in the designs and types of tasks presented to prevent the participant from learning task-specific strategies (Cohen et al., 2024). However, as highlighted by Stone et al. (2023), the most pressing challenge of multiple assessments is the amount of time and effort they require to be completed by the participants (Stone et al., 2023). These factors might influence the willingness of participants to participate, increasing the drop-out rates in research studies, and posing a risk of sampling bias in protocols using EMA. Subjects included in these studies may show differences in sociodemographic and cognitive profiles, as compared with patients who are either unwilling or unable to participate, as well as from those who fail to meet the minimum participation requirements (i.e., subjects who do not complete a minimum number of assessments during the day due to difficulties in utilizing digital devices) (Stone et al., 2023). In addition, the complexity of the EMA impacts the rate of compliance of participants (the ratio of answered prompts in relation to the total number of prompts scheduled) (Moore et al., 2017), due to the high time and effort demand of these multiple assessments (Eisele et al., 2022; Wrzus and Neubauer, 2023). Low compliance rates are of particular importance in EMA, because they pose the issue of how to interpret missing data, which may be the result of a combination of different factors. These include the presence of financial incentives for the patients, the number of prompts, the time interval between prompts, and the intelligence quotient (IQ), age and severity of symptoms of the patients. In addition, missing data may be the result of technical issues (e.g., internet outages, software updates), as well as of “moment selection bias”, i.e., data missed by contextual factors (e.g., participants may avoid EMA assessments in inappropriate or inconvenient situations) or, most importantly, factors directly related to the outcome itself (e.g., if the participant is asked to complete a cognitive task after periods of cognitive fatigue, or in a more distracting environment) (Wu et al., 2021; Stone et al., 2023; Reiter and Schoedel, 2024). This issue is particularly crucial, as data missing because of moment selection bias hinders the ecological validity and generalizability of the assessments (Stone et al., 2023). Lastly, alerts and push notifications for EMA can inadvertently draw attention from individuals near the participants, which could compromise privacy by revealing their participation in a research study. In fact, EMA, as other digital tools, present challenges related to privacy, data security and informed consent, which will be discussed in greater detail in the section “4 Discussion” (Galderisi et al., 2024).

### 3.2 Passive digital phenotyping

Passive DP is an emerging approach in psychiatric research that gathers real-time behavioral data via digital devices, without requiring the active participation of the assessed individual. The acquisition of information in passive DP can involve multiple devices, including not only smartphone sensors (such as Global Positioning Systems (GPS), accelerometers, keystroke dynamics, proximity detectors and ambient light sensors) but also a variety of other wearable devices (e.g., smart watches collecting heart rate variability, skin conductance, and blood pressure) (Khokhlov et al., 2020). DP is gaining traction as a tool for assessing cognitive function in various neuropsychiatric conditions (Cornet and Holden, 2018; Nguyen et al., 2023; Terhorst et al., 2024). Several parameters have been proposed as digital signatures associated with cognitive impairment, such as heart rate, phone usage, sleep data (Chen et al., 2019), keystroke patterns and typing speed (Dagum, 2018; Zulueta et al., 2018), GPS locations (Botros et al., 2022) and gait speed (Rasmussen et al., 2019). In a study by Zulueta et al. (2018) for instance, subjects with bipolar disorder in depressive states showed slower typing speeds and increased autocorrect usage, possibly reflecting impaired attention and self-monitoring, while participants in manic states showed a significant reduction in the use of the backspace key, possibly indicating decreased concentration.

Similarly to EMA, passive DP aims to achieve higher ecological validity by collecting data on cognitive performance in real-world environments. Importantly, it imposes little to no burden on participants, as data collection occurs in the background of the device without requiring the participant’s constant awareness. Thus, in comparison to EMA, DP cognitive assessments are not influenced by the participants’ adherence. In addition, they are less affected by the risks commonly associated with other unsupervised assessments (both computerized cognitive batteries and EMA), such as intentional manipulation of responses (i.e., “cheating”). Additionally, DP offers potentially unlimited temporal resolution, as it can involve the unobtrusive and continuous collection of large volumes of data, thus facilitating highly sensitive and precise assessments of intra-individual variations. This data allows researchers to dynamically adjust the resolution of their analyses — *zooming out*, to identify broader trends over weeks or months; *zooming in*, to explore moment-to-moment fluctuations in relation to specific contexts, times of day, or environmental triggers. This capability may eventually provide unprecedented insights into the temporal and spatial dynamics of cognitive functioning, enabling a more nuanced understanding of psychiatric phenomena.

Despite these significant opportunities, passive DP presents notable challenges. First, the literature on its reliability, validity, and convergence with test-based assessments is still in an early stage of development. Similarly to EMA, methods for estimating and documenting the reliability of passive DP have yet to be fully established. Furthermore, the clinical significance of many proposed digital biomarkers has not yet been thoroughly investigated (Cornet and Holden, 2018; Cohen et al., 2021; Terhorst et al., 2024). Indeed, available studies addressing the convergence between digital biomarkers and clinical ratings often included a low number of subjects, thus limiting the generalizability of their findings (Cornet and Holden, 2018). Several studies report limited or no relationship between passively sensed data and validation measures (Cornet and Holden, 2018; Cohen et al., 2021). These issues pose a significant challenge not only due to the scarcity of studies but also because of remarkable methodological difficulties (Hernandez et al., 2023; Hackett et al., 2024). Indeed, passive DP collects vast amounts of continuous, high-resolution data across diverse temporal and spatial contexts, whereas traditional neuropsychological assessments are typically cross-sectional and designed to assess specific cognitive domains. This discrepancy in resolution makes it difficult to determine the convergence between DP data and standard psychometric evaluations (Cohen et al., 2021). Moreover, similarly to EMA, DP of cognitive performance is challenged by the difficulty of accounting for the numerous contextual variables that influence participants’ daily lives, including environmental noise and external distractors (Hawks et al., 2023). In other words, DP shares with EMA the challenge of the interpretation of both the inter- and intra-individual variability of cognitive performances, and thus the estimation of the reliability of its assessments. The issue is particularly pressing for DP, given the higher risk of misinterpreting the collected digital biomarkers. For example, inactivity detected through GPS may be interpreted as a digital signature of avolition in subjects with schizophrenia, but could be wrongfully interpreted without considering factors like disability or socioeconomic barriers. Finally, issues linked to the feasibility of the use of DP also need to be addressed. In fact, given their strong reliance on the involved digital device, passive DP approaches are particularly susceptible to technical issues, including battery drainage and lack of sensor precision (Cornet and Holden, 2018). In addition, the sheer volume of data which can be collected through passive DP raises practical and logistical issues as well as ethical concerns. Indeed, questions regarding data storage, ownership, protection and access are particularly pressing, especially when collecting highly sensitive data, such as location, behavioral patterns, phone records. Moreover, as the scope of data collection of a study protocol may evolve over time, it becomes essential to ensure that informed consent processes are dynamic and adaptable (Holmlund et al., 2019). Participants must remain fully informed of how their data is being collected, analyzed, and potentially repurposed throughout the study. Addressing these challenges is essential to harness the full potential of passive DP while maintaining ethical and practical standards.

### 3.3 Virtual reality-based assessments

Virtual reality (VR) is a technology that allows users to immerse themselves in a three-dimensional computer-generated scenario (Chirico et al., 2016). VR systems are usually composed of a head-mounted 3D display (HMD), motion-tracking sensors, and controllers. The HMD immerses users in a virtual world, with motion-tracking sensors ensuring the sense of presence by replicating movements and controllers enabling interaction with virtual objects.

The last three decades have seen rapid growth in the application of VR across various clinical domains, including pain management (Rousseaux et al., 2020; Wittkopf et al., 2020; Melillo et al., 2022b; Melillo et al., 2024b), treatment of psychiatric conditions (particularly anxiety-related disorders) (Rus-Calafell et al., 2018; Emmelkamp et al., 2020; Riva et al., 2021; Wiebe et al., 2022), physical rehabilitation (Chirico et al., 2016; Groenveld et al., 2022; Melillo et al., 2022a; Sakdalan and Mitchell, 2023), and for medical and surgical training (Barré et al., 2019). VR can immerse participants in a digital environment, simulating real-world situations, while reducing unwanted sensory stimuli and minimizing many potential confounding variables (Fleming et al., 2023). Consequently, VR environments can also be employed to measure cognitive functioning in settings that are more closely aligned with real-life scenarios while still maintaining controlled and standardized settings (Harvey et al., 2022). In light of this potential, VR in SMI has been tested particularly for the assessment of functional capacity, by measuring performance in the execution of digital simulations of everyday life tasks (Ventura et al., 2020). In relation to this field, tools such as the Virtual Reality Functional Capacity Assessment Test (VRFCAT) have shown good test-retest reliability as well as good convergent validity with traditional measures of functional capacity (Keefe et al., 2016; Harvey et al., 2019). In relation to cognitive assessment, in the last decade, VR has been tested to evaluate specific cognitive domains by administering cognitive tasks embedded within the simulation of more complex real-life activities (Rus-Calafell et al., 2018; Miskowiak et al., 2021). For instance, verbal memory can be assessed by asking participants to recall shopping list items in a VR-replicated supermarket environment. It can also be very efficient to assess spatial memory, as individuals may be asked to remember the correct route to successfully navigate through three-dimensional scenarios (e.g., mazes) (Spieker et al., 2012; Wilkins et al., 2013). Additionally, it has been used to assess executive functioning through tests where participants perform tasks in dynamic environments requiring planning while ignoring distracting stimuli (e.g., running multiple errands in a shopping centre) (Dawson et al., 2009; Kirkham et al., 2024). More in general, VR is particularly interesting as it may help assessing the influence of specific cognitive deficits on real-world functioning, and provide simultaneous assessments of functional capacity and cognitive deficits (Ju et al., 2024). Several VR-based assessment tools have been developed, but the literature on their psychometric properties and particularly on their variability is very sparse (Rus-Calafell et al., 2018; Park, 2022; Kirkham et al., 2024). A recent systematic review on the reliability of VR measures of executive functioning showed that only 5 out of the 19 retrieved trials reported measures of internal consistency for the implemented tool, while none reported an index for test-retest reliability (Kirkham et al., 2024). Similarly, the convergent validity of VR tools, as compared to traditional cognitive tasks, remains to be fully established. A recent meta-analysis by Lee et al. (2024) examined the validity of VR-based tools, as compared to traditional neuropsychological tests for evaluating executive functions, both as a whole cognitive construct or with three subcomponents - cognitive flexibility, attention, and inhibition. The meta-analysis revealed statistically significant positive correlations between scores of VR-based assessments and traditional measures across the three different subcomponents of cognition considered (Lee et al., 2024). However, as highlighted by the authors, the studies presented a high heterogeneity in terms of the correlation effect sizes and showed evidence of publication bias in some of the studies included (Lee et al., 2024). This heterogeneity aligns with findings from studies on other cognitive domains, such as spatial and episodic memory, where research on the validity of VR approaches has also highlighted varying degrees of divergence between VR-based and traditional measures (Miskowiak et al., 2021; Jespersen et al., 2024; Mancuso et al., 2024). Additionally, VR assessment tools often exhibit ceiling effects, limiting their capacity to discriminate different levels of cognitive impairments (Miskowiak et al., 2021).

Beyond validity, a second challenge for VR-based tools concerns their feasibility and acceptability, due to the technological expertise required to use VR systems. This issue may pose a significant barrier for the clinical application of VR for subjects with SMI with cognitive impairment or limited digital proficiency, as it is often the case for people affected by psychiatric disorders, raising potential ethical concerns regarding equity in its use (Garrett et al., 2018). Furthermore, the identification of potentials risks associated to the use of VR in specific categories of subjects with SMI is crucial. Particular attention should be given to the potential long-term effects of VR exposure, such as an increased risk of dissociative symptoms, including derealization and depersonalization (Madary and Metzinger, 2016). To address these risks, the establishment of empirically-driven exclusion criteria is essential, including thresholds on dissociative experience scales to identify individuals who may be particularly vulnerable. Additionally, these factors limit the application of VR for remote assessments, and in clinical trials these are often performed under supervision or following proper training of the participants.

Finally, VR implementation in clinical and research settings is far from achieving consistent integration. As the field is experiencing rapid developments of both the hardware and software components, studies even a year apart are often difficult to compare. This issue is complicated by the fact that often scientific papers do not provide technological specifics, using the term VR to define significantly different technologies (e.g., non-immersive/computer-based and immersive VR), and rarely address theoretical aspects or providing robust theoretical explanations of how VR constructs (e.g., immersivity, tele-presence) apply to the specific areas under investigation (Atkins et al., 2015; Negu? et al., 2016; Garrett et al., 2018; Howard, 2019; Wiener et al., 2020).

Overall, as regard to cognitive assessments, significant challenges still remain in the use of VR, such as creating paradigms that effectively balance precision, ecological validity, and experimental control, while remaining adaptable and applicable to diverse populations and research needs (Krohn et al., 2020).

## 4 Discussion

Digital technologies represent a great opportunity to improve the assessment of cognitive functioning in individuals with SMI, thereby advancing research into their underlying mechanisms and potential treatment options. In the present paper, we aimed to offer an overview of the current research gaps, particularly on issues and possible limitations related to the feasibility, reliability, and validity of these new tools, with the aim of guiding future research and supporting their integration into both clinical practice and research paradigms. The growing interest in these approaches is further driven by their relevance to the longstanding debate between experimental control and ecological validity (Banaji and Crowder, 1989; Conway, 1991; Neisser and Hyman, 2000; Chaytor and Schmitter-Edgecombe, 2003). Indeed, cognitive assessment tools for SMI, traditionally, are designed to maintain high control of the experimental and environmental settings to reduce confounding factors. This approach aims to enhance the reliability and interpretability of the results, providing an accurate reflection of an individual’s cognitive abilities and facilitating the identification of neurobiological mechanisms and potential pharmacological targets (Green et al., 2004). While these methods are generally highly reliable and have good internal validity, they inherently lack ecological validity, as they fail to capture an individual’s true cognitive performance in real-world settings. To address this gap, recently, the FDA has recommended integrating performance-based measures with interview-based approaches capable of gathering insights regarding an individual’s perceived challenges in completing tasks linked to specific cognitive processes (Giordano et al., 2022b). Aligned with this view, differently from traditional cognitive batteries, newer digital approaches aim to enhance ecological validity by capturing cognitive functioning in more naturalistic settings. In relation to EMA and DP, these technologies carry the opportunity of remotely capturing novel data on cognitive functioning in real-world contexts with high temporal resolution. However, accurately assessing naturalistic cognitive performance requires accounting for the near-infinite contextual variables that may influence an individual’s assessment, which in turn are typically controlled in supervised, in-person settings. This is necessary for accurately interpreting both intra-individual and inter-individual variability, as well as their potential causes, which is essential for developing normative data (Holmlund et al., 2019).

In relation to EMA, further research is needed to address remarkable challenges such as participant burden, missing data, sampling biases, moment selection, and the psychometric properties of EMA strategies (Miskowiak et al., 2021; Stone et al., 2023; Lane et al., 2024). In relation to DP, the unique advantage of this methodology is the little to no burden on participants, as data collection occurs in the background of the device without requiring active engagement. This advantage is particularly relevant for schizophrenia since the reduced motivational drive intrinsic to the disorder can affect cognitive performance, highlighting the need for highly engaging yet low-demand tools for both assessment and intervention (Fervaha et al., 2014). Therefore, unlike EMA, DP assessments are less influenced by participant adherence and are not prone to risks such as intentional manipulation of responses. Moreover, DP provides potentially unlimited temporal resolution, enabling unobtrusive, continuous data collection that supports highly sensitive assessments of intra-individual variations. However, the literature on the reliability, validity and convergence with test-based assessments of passive DP cognitive assessments is still in its early stages. Additionally, feasibility issues, including technical challenges and concerns related to data storage, ownership, and protection, need to be addressed in passive DP approaches.

Another key advantage of these new technologies is their ability to operate across virtually unlimited geographical ranges, offering an accessible, highly customizable psychometric method for individuals who might otherwise face significant barriers to traditional assessments. Additionally, although their development may require higher initial financial investments, these technologies could reduce long-term costs by minimizing the need for in-person supervision and thus leveraging scalability. Indeed, evidence on internet- and mobile-based interventions, especially in schizophrenia, highlights their potential to lower healthcare costs, by decreasing the time required from therapists and the need for patient travel, despite higher upfront costs (Kählke et al., 2022). While data on the cost-effectiveness of digital cognitive assessments is limited, their scalability and accessibility suggest they may have a similar cost-saving potential (Griffiths et al., 2006; Hedman et al., 2012; Gómez Bergin and Craven, 2023).

One method that could potentially achieve a balance between the two guiding principles of ecological validity and controlled experimental settings is VR (Parsons, 2015). With the foreseeable future technological advancements, VR has the potential to achieve increasingly higher levels of ecological validity by creating digital scenarios that will be progressively more similar to the ones encountered in real life. At the same time, it allows for strict control over perceptual stimuli and assessment conditions (e.g., number and intensity of stimuli simulating real-world distractors), thus maintaining experimental control and reliability of evaluations. Importantly, VR technologies allow for the assessment of both cognitive functioning and functional capacity, helping to bridge the gap between these two aspects. Furthermore, they offer a platform for both assessment and treatment strategies, thus supporting a more direct translation of cognitive assessments into clinical interventions while ensuring more personalized, feasible, and reliable therapeutic frameworks (Emsley, 2023; Keshavan and Eack, 2023; Schäfer et al., 2023; Sampogna et al., 2024). However, the clinical application of VR paradigms in cognitive assessment will require further study regarding their psychometric properties, tool-specific limitations (e.g., motion sickness) and the thorough development of an underlying theoretical and ethical framework.

The adoption of these technologies raises important ethical considerations, including concerns about privacy and data security. Additionally, there is a need for careful informed consent processes, particularly in the case of extensive data collection methods – such as passive DP – which often have multiple or evolving purposes and destinations. Therefore, it is crucial to establish informed consent processes that are flexible and adaptable to these changes (Couper and Singer, 2013; Kunz et al., 2020; Nica, 2024; Sansone et al., 2024). Keeping in mind the target populations in SMI, balancing autonomy with parental or caregiver involvement remains critical to ensure ethical standards (Kourtis et al., 2019; Lakhtakia et al., 2022; Bartlett, 2024); this aspect is particularly complex in schizophrenia, where individuals affected by the disorder are more likely to lack the capacity to provide informed consent, as compared to healthy control groups, particularly if they present cognitive deficits, as supported by findings from recent meta-analyses (Jeste et al., 2006; Wang et al., 2017; Parsons, 2021). In order to help patients and their legal guardians better understand both the procedures and the potential risks of digital assessment, research could implement interactive tools to assess comprehension (e.g., VR-based demonstrations, digital follow-up questions), ensuring that participants provide fully informed consent. Furthermore, in the case of passive DP approaches, these tools also raise significant concerns about data privacy, protection and storage, as they collect detailed information such as geolocation and keystroke dynamics, potentially exposing sensitive personal details without participants fully understanding how their data will be used (Martinez-Martin et al., 2018; D’Alfonso et al., 2024; Martinez-Martin, 2024; Sansone et al., 2024; Tyano, 2024).

In addition to the previously discussed ethical, VR paradigms introduce tool-specific ethical concerns, including the representation of reality within VR, the autonomy of users, and the potential for unintended effects not only in the virtual setting (*in virtuo*), but also in real life (*in vivo*) (Madary and Metzinger, 2016). For instance, in virtual settings, the anonymity of avatars and lack of accountability can foster harmful behaviors, such as cyber harassment and cyberbullying. These issues, amplified by the immersive nature of VR, may provoke significant emotional distress (Kourtesis, 2024). Additionally, unintended effects of virtual-self representation may extend to real life (*in vivo*), as identification with virtual avatars has been shown to influence real-world behavior, promoting actions consistent with the avatar’s role, whether prosocial or antisocial (Rosenberg et al., 2013). These risks are amplified when VR interventions are used with at-risk groups such as children, the elderly, and individuals with SMI) (Madary and Metzinger, 2016).

This mini-review aimed to provide a brief explorative overview of the potential limitations of current technology-based cognitive assessment, with a particular focus on their feasibility, reliability, and ecological validity. However, some limitations should be noted. First, as mini-reviews prioritize conciseness over comprehensiveness, their scope is necessarily limited, so it may result in the exclusion of relevant studies or perspectives. In particular, we could not discuss all the specific technical issues, implementation requirements and regulatory and legal aspects of the discussed technologies. Furthermore, we decided not to discuss thoroughly data quality control methods and data standardization strategies of the discussed assessment tools. The second limitation is relevant to the narrative review methodology: as our work did not involve an exhaustive systematic search strategy, it may have potentially excluded some relevant studies. Furthermore, our work did not involve either a quantitative analysis of the retrieved evidence or a systematic assessment of the quality of included studies, as these were beyond the scope of our review. However, this work might serve as a basis for future quantitative systematic reviews and meta-analyses addressing these research questions and evaluating the robustness and quality of the associated evidence.

## 5 Conclusion and future perspectives

Technology-based assessments can provide valuable insights into cognitive impairment and serve as a complementary tool to traditional cognitive evaluations. By bridging the gap between cognitive performance measured in controlled experimental settings and real-world cognitive functioning, these technologies can significantly enhance our understanding of how cognitive impairments impact functional outcomes. This, in turn, may crucially contribute to the development of more effective and personalized cognitive and functional rehabilitation programs, ultimately addressing the disability burden associated with severe mental illnesses (Palumbo et al., 2022; Giuliani et al., 2024; Perna et al., 2024).

Addressing the challenges discussed in the present paper is therefore essential to ensure that these emerging technologies achieve these goals.

In relation to EMA and passive DP, a promising path forward may lie in the combined application of these tools. A successful example is the collection of passive DP data during EMA assessments (i.e., “paradata”). Paradata, as applied for this context, refers to auxiliary data collected during the administration of digital assessments (e.g., response times, navigation patterns, position). This approach may be particularly promising, as it may contribute to the development of more “context-aware” EMA assessments, by using digital devices to capture observations of both the individual and its environment (Burns et al., 2011). These data can help researchers infer aspects of cognitive performance or identify factors affecting data quality and validity. In addition, as DP paradata can provide data beyond the primary scope of the EMA assessment, it can provide more multidimensional observations of the participant. For example, researchers can analyze behavioral metrics like reaction times during survey responses to infer cognitive performance, even if the primary focus of the survey is on other dimensions, which may be of interest for the evaluation of cognitive functioning (e.g., questions evaluating mood or anxiety) (Dagum, 2018; McClain et al., 2019; Abi-Dargham et al., 2023; Berk, 2023; Hernandez et al., 2023; Tamminga, 2023; Voineskos, 2023). Thus, the combination of passive DP and EMA can improve the ecological validity of the assessments by providing context-aware and multidimensional observations. Given the high dependence on the precision of the employed applied digital devices, further studies should contribute to the development of standardized data quality control methods and algorithms (Cornet and Holden, 2018). In relation to VR, the development of robust theoretical frameworks for VR constructs will be critical for the study of the psychometric properties of VR-based evaluations. In addition, more detailed reporting of technological specifications is essential to enhance the comparison of study results and to support efforts toward standardization. Overall, further studies are needed to broaden the evidence base on the validity, feasibility and psychometric properties of these evaluation tools. Particularly in the case of EMA and DP, it is urgent to develop and implement improved reliability estimation methods (Stone et al., 2023). Future studies should always report relevant reliability and variability coefficients in order to enhance transparency and to inform future study design decisions. Future implementations should focus on improving accessibility for patients with varying levels of digital literacy. For instance, providing pre-configured devices or step-by-step guidance for patients and caregivers could help ensure more equitable access to these technologies. Importantly, there is a lack of a unified ethical and regulatory framework for the implementation of digital technologies in mental health care (Steindl, 2023; Galderisi et al., 2024). This is a crucial issue particularly in relation to EMA and DP, as the development of standardized frameworks for secure data handling and storage is a necessary step for their safe and ethical implementation.

Finally, as current studies are predominantly conducted in high-income countries, further research will need to involve cross-cultural validation and investigate the adaptability and feasibility of these tools in varied socio-economic and cultural contexts (Moore et al., 2022; Hernandez et al., 2023; Hackett et al., 2024; Harmon et al., 2024; Henneghan et al., 2025).
