# Supplementary material for: Current limitations in technology-based cognitive assessment for severe mental illnesses: a focus on feasibility, reliability, and ecological validity
Source: Front Behav Neurosci. 2025 Apr 7;19:1543005. doi: 10.3389/fnbeh.2025.1543005 (PMC12009854; doi:10.3389/fnbeh.2025.1543005)
Supplement: Supplementary file 1 [file Table_1.docx]

**Supplementary Material**

**S1. Methods**The present study is a narrative review aimed at synthesizing key findings on technology-based cognitive assessments in severe mental illness, identifying methodological considerations, feasibility aspects, and ethical challenges. Unlike systematic reviews, which seek exhaustive coverage of a research domain, narrative reviews prioritize conceptual depth and theoretical contributions. A structured search was conducted across PubMed, Scopus, and PsycINFO, supplemented by manual screening of reference lists and relevant grey literature. Search terms included constructs related to cognitive impairment (e.g., "executive function," "working memory," "processing speed," "social cognition") and psychiatric conditions (e.g., "schizophrenia," "bipolar disorder," "major depressive disorder"), cross-referenced with digital assessment methods (e.g., "ecological momentary assessment," "experience sampling," "digital phenotyping," "virtual reality"). Studies were included if they examined digital cognitive assessments in psychiatric conditions, with a primary focus on feasibility, validity, or ethical considerations. Studies were excluded if they did not involve digital tools, were theoretical papers or commentaries without empirical data, or were not published in English. Given the heterogeneity of the included studies, findings were synthesized qualitatively, highlighting methodological implications rather than performing a formal meta-analysis.
